# Effects of calcium on cell wall metabolism enzymes and expression of related genes associated with peel creasing in *Citrus* fruits

**DOI:** 10.7717/peerj.14574

**Published:** 2022-12-20

**Authors:** Bin Huai, Yunli Wu, Chunhui Liang, Panfeng Tu, Tingting Mei, Anquan Guan, Qing Yao, Juan Li, Jiezhong Chen

**Affiliations:** 1 South China Agricultural University, Guangzhou, China; 2 Guangdong Agriculture Industry Business Polytechnic College, Guangzhou, China; 3 Zhongkai University of Agriculture and Engineering, Guangzhou, China; 4 Lianjiang Fruit Development Center, Lianjiang, China

**Keywords:** Citrus, Calcium, Cell wall metabolism, Fruit creasing

## Abstract

Fruit peel creasing is a serious pre-harvest physiological disorder in citrus, influencing fruit quality, storage, and yield. Four- and eight-year-old ‘Hongjiang’ oranges grafted onto Canton lemon rootstocks were treated with calcium and calcium inhibitors, respectively, to study the effects of different treatments on fruit creasing rate, mechanical properties of the peel, cell wall metabolism enzyme activities, and the expression of related genes. Foliar application of 0.5% calcium nitrate significantly reduced the fruit creasing rate, while treatment with EGTA and LaCl_3_, inhibitors of calcium uptake, increased the fruit creasing rate; But the effect of calcium nitrate treatment on changing the mechanical properties of pericarp and inhibiting the activity of hydrolase (PG, Cx and PE) was not very significant. Furthermore, it was observed that the expression levels of genes (*PG*, *Cx*, and *PE*) encoding cell wall-degrading enzymes were significantly lower in the normal fruit peel than in the creased fruit peel. Meanwhile, the expression levels of *PG*, *Cx*, and *PE* were higher in the peel of shaded fruit than in the peel of exposed fruit. During the high incidence period of fruit creasing, calcium nitrate treatment down-regulated the expression of *PG*, *Cx*, and *PE*, while EGTA treatment up-regulated the expression of these genes. In conclusion, foliar spraying of calcium nitrate at the fruit rapid enlargement stage can increase the Ca content in the peel of ‘Hongjiang’ orange and significantly suppress the expression of cell wall degrading enzymes genes (*PG*, *PE* and *Cx*) in ‘Hongjiang’ orange peel during the high occurrence period of fruit creasing, resulting in reducing the occurrence of fruit creasing and cracking.

## Introduction

Fruit creasing or cracking has been found in many *Citrus* varieties, such as ‘Shatangju’, ‘Kara’ oranges, and ‘Hongjiang’ oranges, during the ripening of the fruit. Fruit creasing occurs due to the separation of cells in the middle lamella of albedo tissue, resulting in creases on the surface of the fruit, or even fruit cracking (Li et al., 2009; Li & Chen, 2017). This disorder affects its yield and storage quality resulting in substantial economic losses to growers (Li & Chen, 2017). The incidence of creasing or even cracking is known to be influenced by multiple factors such as external factors including agronomic (irrigation, rootstock selection, mineral nutrition and plant growth regulators) and environmental factors (climate, high humidity and rainfall), fruit characteries (fruit shape and size, sugar accumulation and mechanical properties of the peel (peel thickness and hardness) (Gambetta et al., 2000; Treeby & Storey, 2002; Bower, 2004; Li et al., 2009; Tam, Singh & Behboudian, 2012; Khadivi-Khub, 2014; Li & Chen, 2017; Correia et al., 2018).

Previous studies have shown that the incidence rate of fruit creasing is remarkably lower in fruits growing on the sunny side of trees or around tree crowns than in fruits growing on the shady side of trees or inside tree crowns. In addition, fruit creasing and cracking usually occurs on the shaded sides of fruits (Gambetta et al., 2000; Treeby et al., 2000; Li & Chen, 2017). Current studies on the molecular mechanism about fruit creasing or cracking have focused on the proteins that are involved in cell wall disintegration or hydrolysis during plant cell expansion (Lu et al., 2006; Wang et al., 2006; Kasai et al., 2008; Balbontín et al., 2014; Li et al., 2014; Yu et al., 2020; Wang et al., 2021). These proteins related to cell wall metabolism including Polygalacturonase (PG), cellulase (Cx), pectinmethylesterase (PE), expansin (EXP), pectate lyase (PL), xyloglucan endotransglycosylase (XET) and β-galactosidase (β-Gal) (Lu et al., 2006; Wang et al., 2006; Kasai et al., 2008; Wen & Shi, 2012; Balbontín et al., 2014). High activity levels of PG, Cx, and PE could promote the degradation of cell walls, which is related to fruit cracking (Roberts et al., 2000; Yang et al., 2016; Li & Chen, 2017). Also, Giné-Bordonaba et al. (2017) found cherry cracking sensitivity is associated with cell wall modifying enzymes. Previous studies have revealed that PG, PE, and Cx are significantly more active in the pericarp of ‘Hongjiang’ oranges than in the pericarp of ‘Anliu’ oranges which are resistant to creasing (Li et al., 2009).

Fruit creasing or cracking is not regulated by a single gene, but regulated by a series of genes. Li et al. (2009) reported lower expression of *Ct-Exp1* in ‘Hongjiang’ oranges, which have a higher incidence rate of fruit creasing than ‘Anliu’ oranges. Kasai et al. (2008) discovered that the different expression of *MdEXPA3* in exocarps and mesocarps could be a possible cause of interior ring cracking in apple. In addition, the expression of *LcExp1* and *LcExp2* genes in lichi peel was closely related to fruit cracking (Wang et al., 2006). Lu et al. (2006) found that cracking was closely related to the differential expression of *LcXET1* in the pericarp and pulp in ‘Huaizhi’ lychees, which were unsusceptible to cracking, and ‘Nuomici’ lychees, which were susceptible to cracking. In addition, NAA treatment increased the accumulation of *LcXET1* mRNA in the pericarp of ‘Nuomici’ lychees, then reducing fruit cracking. Chen et al. (2019) found that pectinesterase (PE) and PG were related to the pectin degradation of atemoya peel, which can affect fruit ripening and cracking. Recently, different omics studies play a important role in understanding fruit cracking (Chen et al., 2019; Xue et al., 2020; Zhu et al., 2020). A large number of genes or protein related to fruit cracking have been screened out. These genes or protein were involved cell wall metabolism, water transport, plant hormones metabolism (auxin, gibberellins, ethylene and abscisic acid), calcium transport and calcium-binding protein including calmodulin proteins (CaM) which play a cruial role in both regulating plant growth and development (Li et al., 2014; Zhu et al., 2020; Shi & Du, 2020; Wang et al., 2021). However, few studies have been conducted on the molecular mechanisms behind *Citrus* fruit creasing and cracking, and the concrete molecular mechanism about fruit creasing or cracking remains unclear.

Currently, most of the studies on the prevention of cracking have been based on foliar treatments with mineral elements. One of the foliar treatments used most to reduce cracking is calcium (Ca) application. Its effectiveness has been demonstrated in different types of crops such as litchi, pomegranate, sweet cherry or watermelon (Davarpanah et al., 2018; Winkler & Knoche, 2019; Lopez-Zaplana et al., 2020; Michailidis et al., 2020, 2022). Foliar application of calcium fertilizer significantly reduces fruit creasing and cracking rate in *Citrus* (Treeby & Storey, 2002; Tam, Singh & Behboudian, 2012; Bakshi, 2018). Calcium treatment significantly increases the Ca^2+^ content of the cell wall, particularly the bound calcium content (Alvaro, Victoria & Jesús, 2010). In addition, calcium treatment attenuates the activities of pectinase, cellulase, pectin methoesterase, β-galactosidase, and xylanase in fruit pericarps, while increasing levels of pectin, hemicellulose, and cellulose, which modify the components of the cell wall, increase the thickness and hardness of fruit pericarps and decrease fruit creasing rate (Li et al., 2009; Li & Chen, 2017). To date, few studies have investigated the role of calcium in cell wall modification in *Citrus* fruit pericarps. This study aims to analyze the effects of calcium on fruit creasing rate, fruit peel cell wall-modifying enzymes, and related gene expression levels in ‘Hongjiang’ oranges by spraying Ca(NO_3_)_2_ and calcium-absorption inhibiting agents on the plants. In addition, the molecular mechanisms behind reduction of fruit creasing by foliar spraying of calcium nutrients has also been explored, which has important theoretical guiding significance for the prevention of *Citrus* fruit cracking and creasing and improving fruit quality.

## Materials and Methods

### Plant materials and experimental design

Eight- and four-year-old ‘Hongjiang’ oranges (*Citrus sinensis* (L.) Osbeck cv Hongjiangcheng) were used as experimental materials and conducted in Hejiangqiao Orchard, Lianjiang City, Guangdong Province in 2014 and 2015 respectively. Foliar spray treatments were performed on July 1 and July 20 in 2014, and on June 10 and June 25 in 2015 at the fruit rapid enlargement stage, respectively. The experiment consisted of the following five treatments: (1) 0.5% Ca(NO_3_)_2_; (2) 2.5 mmol L^−1^ LaCl_3_ (a plasma membrane Ca^2+^ channel blocker); (3) 5 mmol L^−1^ ethylene glycol tetraacetic acid (EGTA) (an extracellular Ca^2+^ chelating agent); (4) 0.5 mmol L^−1^ trifluoperazine (TFP, a CaM anticoagulant); and (5) a control group, CK (water). The solution was sprayed on the surface of the leaves and fruit over the whole tree until it drips. Each tree was sprayed with about 5 L of solution. The treatments and control were performed with five biology repications (a tree per replication). Identical irrigation and fertilization protocols were applied to each treatments. Eight fruits without signs of disease and similar size including four outside and four inside of the canopy were picked from each tree every 30 days from Aug. 15 to Dec. 1, 2014 and from July 27 to Nov. 27, 2015 (the fruits were picked from immature to ripe). These fruits were promptly put in a thermal container and brought back to the laboratory. After washing with distilled water, a portion of clean and smooth pericarp was taken and was frozen in liquid nitrogen before being stored in a refrigerator at −80 °C. Some of these samples were used to test cell wall metabolism enzymes, while others to test the expression of genes encoding cell well metabolism enzymes.

### Investigation of the incidence of fruit creasing and determination of mechanical properties of peel

To test the incidence of fruit creasing, the number of fruit per plant (C_1_) and the total number of creasing fruit (C_2_) were counted, and the fruit creasing rate was calculated as follows: C_0_ (%) = (C_2_/C_1_) × 100. The skin hardness was measured using an Instron 5542 single-column benchtop electronic universal material testing machine manufactured by Intermec, Inc. The probe was circular in shape and the probe diameter was 2 mm. The probe moving speed was 400 mm/min. The maximum pressure during the pressing process was taken as the fruit hardness value in Newtons (N). The different parts of each fruit were measured three times and averages were calculated. The thickness of the peel was measured using a Mitutoyo digital caliper (Model: 500–673), and each fruit was evenly cut into four portions. The peel was completely peeled off and the middle parts of the fruit was measured as the total peel thickness T (Unit: mm), peel thickness was then calculated using the following equation: t = T/4 (unit: mm).

### Determination of Ca content in pericarp

The content of Ca in peel was determined according to Lu et al. (2013) with some modifications. Separated peel were washed with distilled water and then dried at 105 °C for 30 min and then were dried at 70 °C to constant weight. 200 mg sample was incinerated in a muffle furnace (550 °C for 6 h). and the ash was dissolved in 2 mL HCl. The content of Ca were measured by atomic absorption spectrometry.

### PG, Cx, and PE activity assays

The cell wall metabolic enzyme solution was extracted following the method of Pathak, Mishra & Sanwal (2000). The peel of ‘Hongjiang’ orange were homogenized in 0.05 mol L^−1^ citric acid phosphate buffer (pH5.5, 1 M NaCl, 10 g L^−1^ PVPP and 0.6% EDTA) under ice-cold conditions for 3 h. The solution was then centrifuged at 10,000*g* for 30 min at 4 °C, and the supernatant was used for enzyme activity assay. Cellulase (Cx) activity assay: 0.5 mL extractive of sample was incubated in 2 mL rection buffer (pH5.2, 0.05 mol L^−1^ citric acid phosphate buffer containing 1 M NaCl, 0.6% EDTA and 1% sodium carboxymethylcellulose) at 40 °C for 60 min. And the reaction was terminated by adding 1 mL DNS. Then the sample was immediately put into boiling water for 5 min, and then quickly cooled with running water. OD_540_ was measured using a spectrophotometer. Glucose was used to make the standard curve. Polygalacturonase (PG) activity assay: 1 mL extractive of sample was incubated in 2 mL rection buffer (pH4.0, 0.05 mol L^−1^ citric acid phosphate buffer containing 2% pectin) at 37 °C for 30 min. And the reaction was terminated by adding 1 mL DNS. Then the sample was immediately put into boiling water for 5 min, and then quickly cooled with running water. OD_520_ was measured using a spectrophotometer. Galacturonic acid was used to make the standard curve. NaOH titration (Abu-Goukh & Bashir, 2003) was employed to measure pectinesterase (PE) activity, with some modifications. A total of 2 mL enzyme solution was taken and pre-heated at 37 °C for 3 min, then three drops of phenolphthalein indicator were added as well as 4 mL of 1% *Citrus* pectin substrate. After that, 0.1 mol L^−1^ NaOH was used to titrate until the solution became light red, then it was incubated at 37 °C for 30 min, during which NaOH was periodically added to neutralize the pectin acid generated in the process. The total volume of NaOH used to keep the solution light red was recorded. The amount of NaOH was then calibrated with potassium hydrogen phthalate. One micromole of CH_3_O^−^ released by pectin catalyzed per minute per gram of fresh sample under the abovementioned conditions was utilized as an enzyme activity unit (U).

### Quantitative RT-PCR

A Plant RNA Extraction Agent Kit supplied by Beijing Huayueyang Corporation was adopted to extract total RNA from fruit pericarps. The first strand of cDNA was synthesized using the superscript first-strand synthesis system (Invitrogen, Waltham, MA, USA). Gene expression were detected by qRT-PCR and primers were designed using Primer premier 5.0 software (Table 1). Gene sequence were obtained from http://citrus.hzau.edu.cn/cgi-bin/orange/search. The NCBI Blast tool was used to check the specificity of primers in the *Citrus* genome database, and Shanghai Bioengineering Co, Ltd. synthesized the primers. *18SrRNA* was selected as the reference gene. Analysis of gene expression was conducted according to instructions of iTaq^TM^ Universal SYBR Green Supermix, manufactured by Bio-Rad corporation using CFX96 Real-Time System (Bio-Rad, Hercules, CA, USA). The 20 μL reaction system comprised 5 μL of the cDNA template, 0.5 μL each of the upstream and downstream primers, 10 μL of reaction MIX, and 4 μL of sterile ddH_2_O. The reaction procedure followed the following two-step method: 95 °C, 30 s; 95 °C, 5 s, 60 °C, 30 s, for a total of 40 cycles and with sterile ddH_2_O as a negative control. The relative expression levels of genes were calculated using the 2^−∆∆Ct^ method (Livak & Schmittgen, 2002), and each value was an average of the values of three biological replicates.

**Table 1:** Primers for the qRT-PCR. *18S rRNA* is reference gene.

Accession number	Gene name	Primer sequence (5′–3′)
FJ356261.1	*18SrRNA*	F-ACGATTCCACAGATCGAGGT
		R-GGCAAAATCAAGAGTTCCA
NM_001288930	*PE*	F-TCCCATTCTCCCTTGGTTTG
		R-GAAGCCACGAATAAGACACG
NM_001288908	*PG*	F-CTTAGGGTGGTCAACAGT
		R-CCGTATTAGGACTTTCAGC
NM_001288880	*Cx*	F-TGTGGCAATATGGTAGTCAA
		R-CCAATGAGGGTAAAGAAGAT

### Statistical analyses

*t*-tests and Duncan multiple comparisons were performed using the SPSS21.0(IBM) statistical software.

## Results

### Effect of different treatments on fruit creasing rate of ‘Hongjiang’ oranges

**Figure 1:**
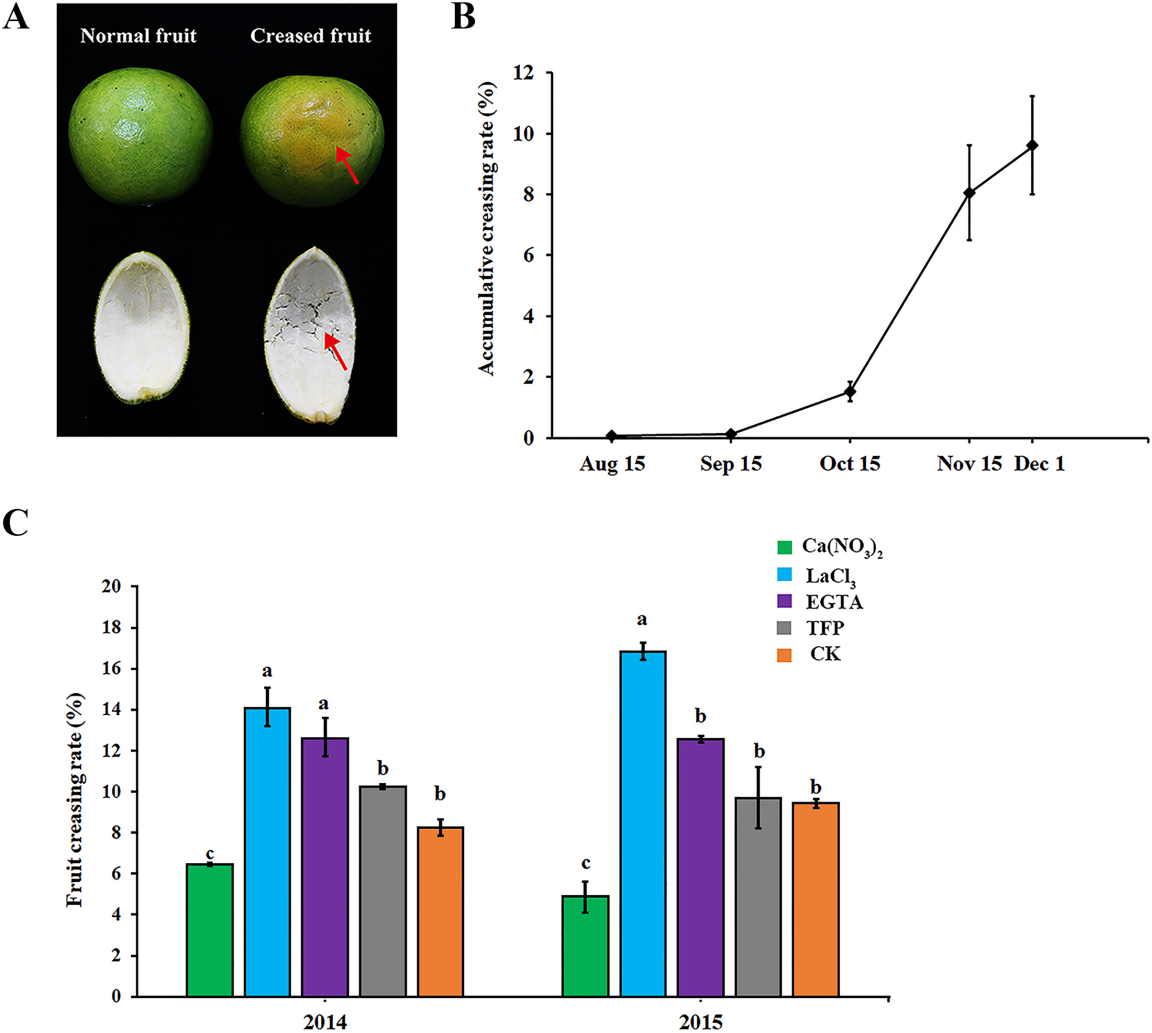
Effect of different treatments on fruit creasing rate of ‘Hongjiang’ oranges. (A) The image of normal fruit and creased fruit of ‘Hongjiang’ orange. The surface of the normal fruit is smooth, whereas the creased fruit exhibits subsidence of the epidermis layer, irregular grooves on the fruit surface, and separations of cells in the middle peel (red arrows). (B) Accumulative fruit creasing rate of ‘Hongjiang’ oranges in 2014. (C) Effect of different treatments on fruit creasing rate of ‘Hongjiang’ oranges in 2014 and 2015. Different lowercase letters indicate a significant difference between the treatments at *p* < 0.05 (Duncan’s multiple range test). The values are the means ± SEs (*n* = 3).

As shown in Fig. 1A, ‘Hongjiang’ orange creased fruits were mainly characterized by the subsidence of the epidermis layer, irregular grooves on the fruit surface, and the separation of cells in the middle pericarp (Fig. 1A, red arrow). In 2014, fruit creasing began to occur in middle and late September, and the cumulative incidence of fruit creasing increased by 6.5% from Oct. 15 to Nov. 15. However, the cumulative incidence of fruit creasing only increased by 1.5% from Nov. 15 to Dec. 1 (Fig. 1B). Thus, we thought that the period from Sep. 15 to Dec. 1 is the occurrence period of fruit creasing during the growth of ‘Hongjiang’ orange, and especially from Oct. 15 to Nov. 15 is the highest occurrence period of fruit creasing.

The results of the two field experiments in 2014 and 2015 suggested that foliar application of calcium and calcium inhibitors had a certain effect on the cumulative incidence of fruit cracking in ‘Hongjiang’ orange, with similar effects observed for each treatment across the 2 years (Fig. 1C). The cumulative fruit creasing rate of ‘Hongjiang’ oranges treated with calcium nitrate was the lowest among all treatments across the 2 years, and was significantly lower than that of the control (CK). Meanwhile, the fruit creasing rate of ‘Hongjiang’ oranges treated with LaCl_3_, which is a plasma membrane Ca^2+^ channel blocker, was the highest among all treatments across the 2 years and was significantly higher than that of the control (CK). This was followed by the EGTA treatment, which was significantly higher than the control in 2014. The incidence of fruit creasing in oranges treated with TFP, a kind of CaM inhibitor, was no significant difference from that of the control in both 2014 and 2015.

### Effects of calcium and calcium inhibitor treatments on mechanical properties of ‘Hongjiang’ orange peel

**Figure 2:**
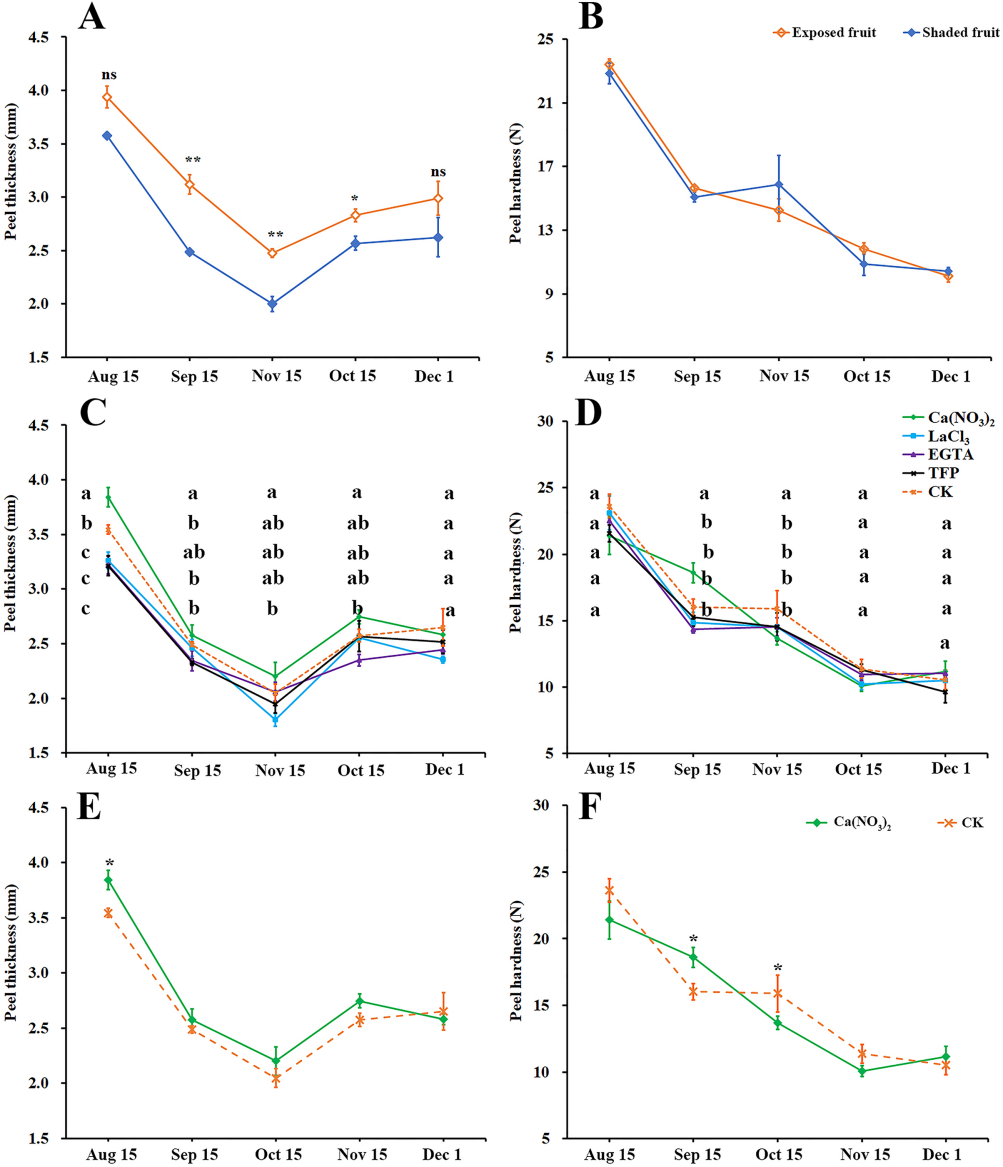
Effects of calcium and calcium inhibitor treatments on mechanical properties of ‘Hongjiang’ orange peel. (A, B) Comparison of thickness and hardness of fruit peels between exposed fruits and shaded fruits of ‘Hongjiang’ orange. (C, D) Effects of calcium and calcium inhibitor treatments on fruit peel thickness and hardness in ‘Hongjiang’ oranges. (E, F) Comparison of thickness and hardness of fruit peels between under calcium treatment and under the control. The values are the means ± SEs (*n* = 3); Different lowercase letters indicate a significant difference between the treatments at *p* < 0.05 (Duncan’s multiple range test); Asterisks (* and **) indicate significant difference between calcium treatment and control at *P* < 0.05 and *P* < 0.01 level, respectively (t-Student test).

Peel thickness of ‘Hongjiang’ oranges first decreased and then increased (Fig. 2A). The thickness of exposed fruits was greater than that of shaded fruits from Sep. 15 to Nov. 1, with the difference reaching an especially significant level between Sep. 15 and Oct. 15. As the fruits ripened, the peel gradually softened, but there was no significant difference in peel hardness between the exposed fruits and shaded fruits (Fig. 2B).

This research also determined the effects of calcium and calcium inhibitor treatments on the thickness and hardness of ‘Hongjiang’ orange fruit peel (Figs. 2C–2F). The results suggested that the fruits treated with calcium nitrate had significantly thicker peels than that of the control on Aug. 15 (Figs. 2C, 2E), while fruits treated with LaCl_3_ and TFP had significantly thinner peels than that of the control on Aug. 15 (Fig. 2C). The hardness of the peel gradually decreased with the ripening of the fruit (Figs. 2D, 2F). Fruits treated with calcium nitrate had significantly harder peels than that of the control on Sep. 15. There were no significant differences between other treatments and the control (Fig. 2D).

### Effects of calcium and calcium inhibitor treatments on the Ca content in ‘Hongjiang’ orange peel

**Figure 3:**
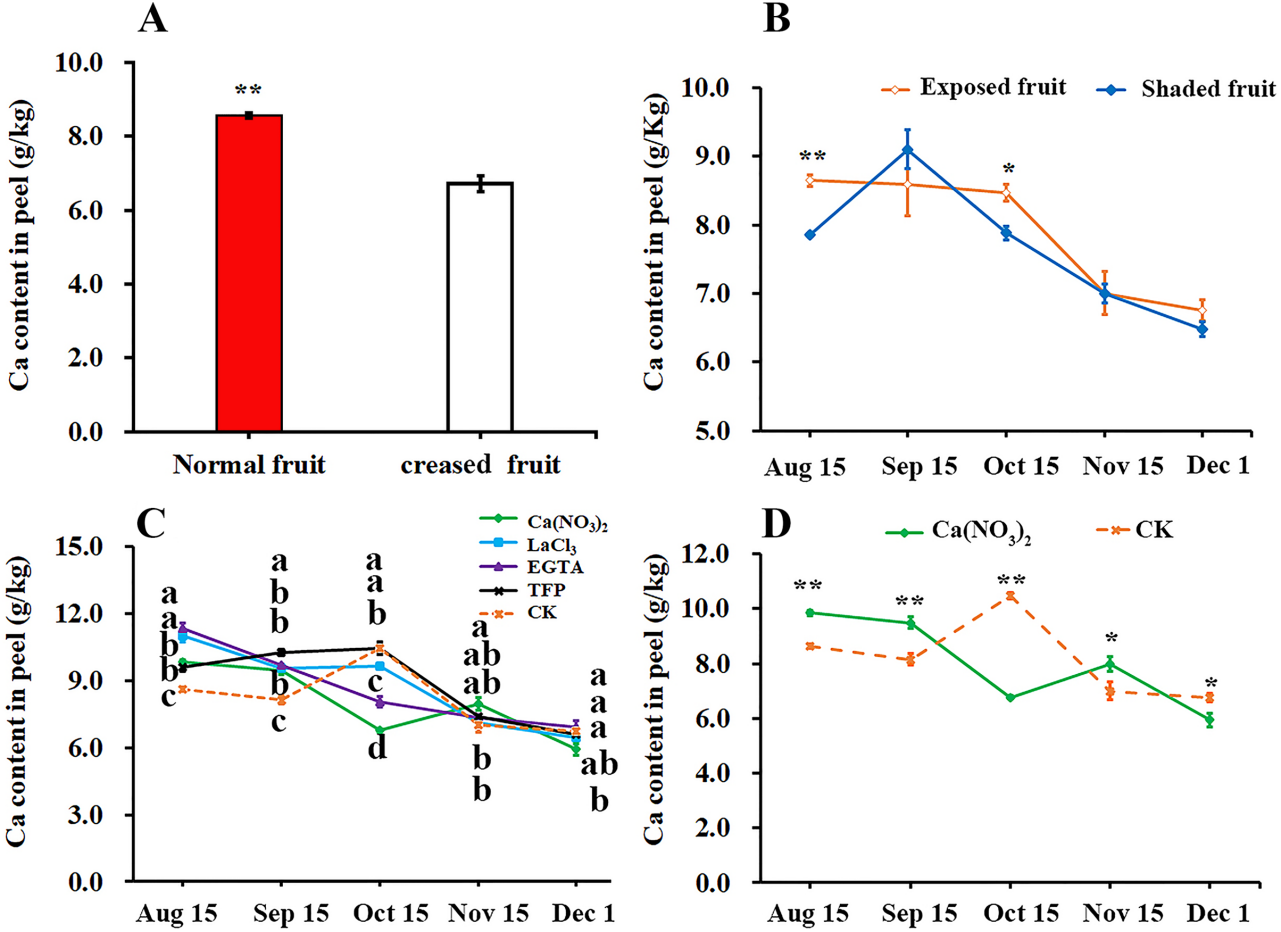
Effects of calcium and calcium inhibitor treatments on the calcium content in ‘Hongjiang’ orange peel. (A) The calcium content in the peel of normal fruit and creased fruit. (B) The calcium content in the exposed fruit and shaded fruit. (C) The calcium content in the peel under different treatments and control. (D) The calcium content in the peel under calcium treatment and control. The values are the means ± SEs (*n* = 3); Different lowercase letters indicate a significant difference between the treatments at p < 0.05 (Duncan’s multiple range test); Asterisks (* and **) indicate significant difference between calcium treatment and control at *P* < 0.05 and *P* < 0.01 level, respectively (t-Student test).

We compared the difference of calcium content in peel between the normal fruit and creased fruit and found that the calcium content in the normal fruit peel was significantly higher than that in creased fruit peel of ‘Hongjiang’ orange (Fig. 3A). Furthermore, we found that the calcium content in exposed fruit peel of ‘Hongjiang orange’ was significantly higher than that of shaded fruit on Aug. 15 and Oct. 15 (Fig. 3B). Also we determined the effect of different treatments on the calcium content in the peel and found that the calcium content in the peel of each treatment decreased from Aug. 15 to Dec. 1 in general, but the calcium content in the pericarp increased slightly from Sep. 15 to Oct. 15 under control, TFP and LaCl_3_ treatments (Fig. 3C). Interestingly, Compared with the control, the period of calcium content increased in pericarp treated with calcium was delayed. Calcium content in the peel of ‘Hongjiang orange’ treated with calcium nitrate spraying increased from Oct. 15 to Nov. 15, while the calcium content in the peel treated with EGTA decreased from Aug. 15 to Dec. 1. The calcium content in the peel treated with calcium nitrate increased first and then decreased from Oct. 15 to Dec. 1, and the decrease was only 22.31% of that of the control. The calcium content in the peel treated with calcium nitrate was significantly higher than that of the control on Nov. 15 when is the highest occurrence period of fruit creasing during the growth of ‘Hongjiang’ orange. However, the content of calcium in peel with LaCl_3_, EGTA, TFP and control treatment decreased significantly from Oct. 15 to Dec. 1 (Figs. 3C, 3D).

### Effects of calcium and calcium inhibitor treatments on the activities of cell wall metabolic enzymes in ‘Hongjiang’ orange peel

**Figure 4:**
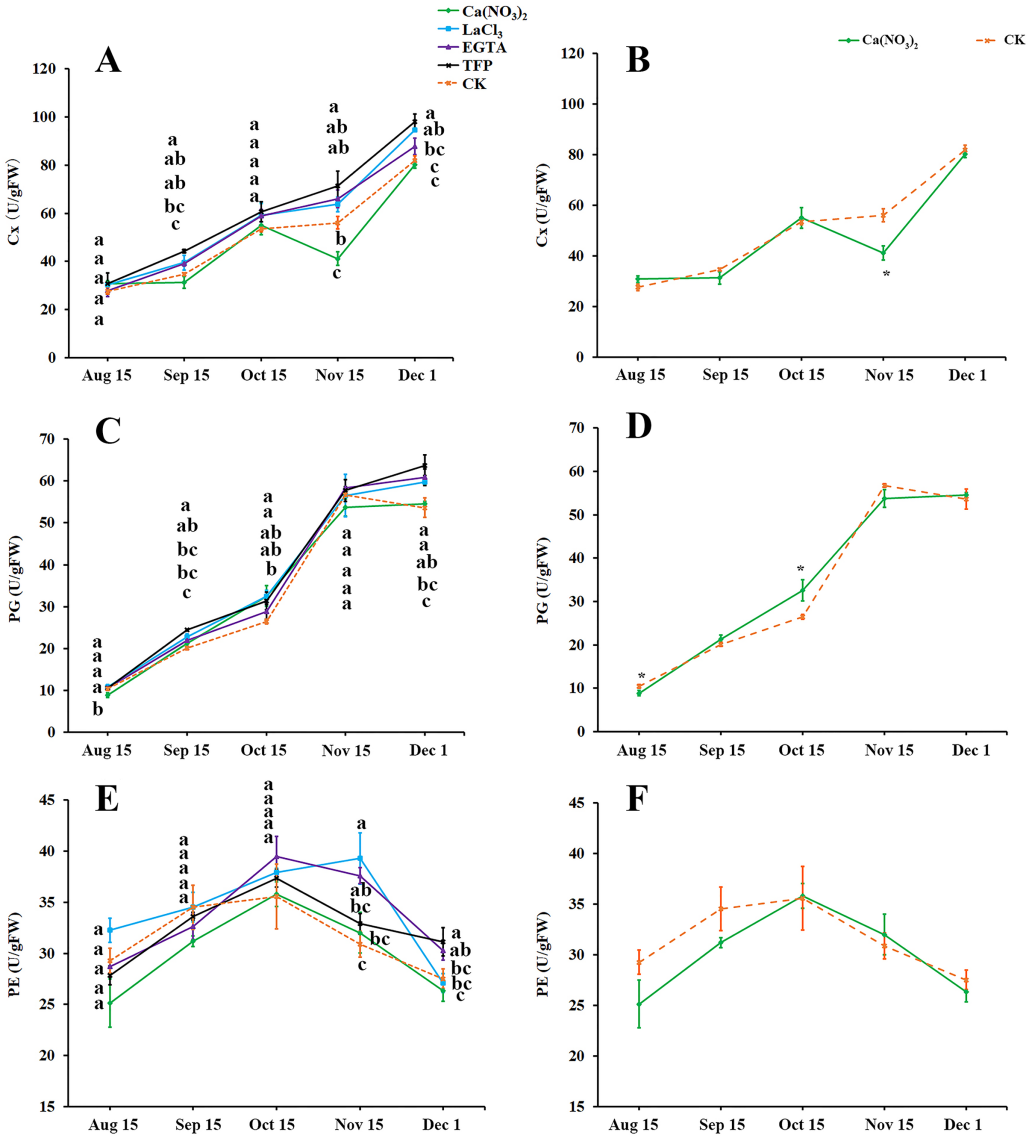
Effects of calcium and calcium inhibitor treatments on the activities of cell wall metabolic enzymes in ‘Hongjiang’ orange peel. (A, B) Effects of calcium and calcium inhibitor treatments on the Cx activities in ‘Hongjiang’ orange peel. (C, D) Effects of calcium and calcium inhibitor treatments on the PG activities in ‘Hongjiang’ orange peel. (E, F) Effects of calcium and calcium inhibitor treatments on the PE activities in ‘Hongjiang’ orange peel. The values are the means ± SEs (*n* = 3); Different lowercase letters indicate a significant difference between the treatments at *p* < 0.05 (Duncan’s multiple range test); An asterisks (*) indicates significant difference between calcium treatment and control at *P* < 0.05 level (t-Student test).

As fruit creasing in oranges occur mostly in the canopy (Gambetta et al., 2000; Treeby et al., 2000), this research determined the effect of calcium and calcium inhibitor treatments on the activities of cellulase (Cx), Polygalacturonase (PG), and Pectinesterase (PE) in the pericarps of ‘Hongjiang’ oranges growing inside the canopy (Figs. 4A–4F). The activity of Cx gradually increased along with the development of the ‘Hongjiang’ oranges. Cx activity was lower in peel treated with calcium nitrate than in the control on Nov. 15, while the Cx activity in peel treated with LaCl_3_ was significantly higher than that of the control on Dec. 1. And the activities of Cx in the pericarps treated with TFP were significantly higher than that of the control on Sep. 15, Nov. 15 and Dec. 1 (Figs. 4A, 4B). The activity of PG increased along with the development of the fruit (Figs. 4C, 4D). PG activity was significantly lower in peel treated with calcium nitrate than in the control on Aug. 15. PG activity was significantly higher in the TFP-treated peel than that in the control on Sep. 15 and Dec. 1. PG activity was significantly higher in LaCl_3_-treated peel than that in control on Sep. 15, Oct. 15 and Dec. 1 (Figs. 4C, 4D). PE activity in the pericarp during the development of the shaded fruits peaked on Oct. 15, but it peaked on Nov. 15 under the LaCl_3_ treatment. And PE activity was significantly higher in peel treated with EGTA or LaCl_3_ than in the control on Nov. 15. PE activity was significantly higher under the TFP treatment than under the control on Dec. 1, while the calcium nitrate treatment did not differ significantly from the control (Figs. 4E, 4F).

### Effects of calcium and calcium inhibitor treatments on the expression of metabolic enzyme genes in ‘Hongjiang’ orange cells

**Figure 5:**
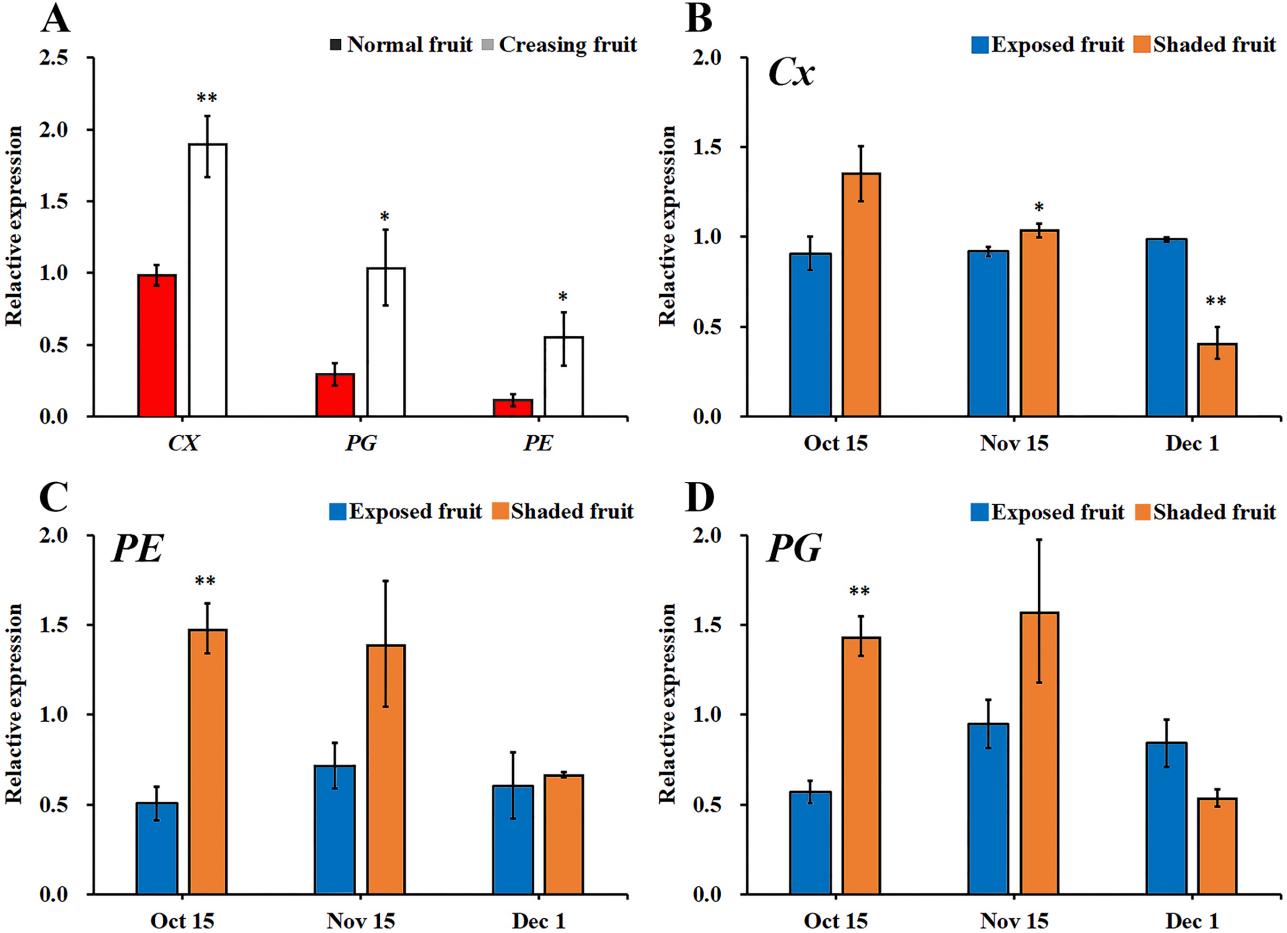
Comparison of gene expression of metabolizing enzymes in different types fruit peel of ‘Hongjiang’ orange. (A) Comparison of gene expression of metabolizing enzymes between normal fruit peel and creased fruit peel in 2014. (B–D) Comparison of the expression of *Cx*, *PE*, and *PG* between shaded fruit peel and exposed fruit peel in 2014. The values are the means ± SEs (*n* = 3); Asterisks (* and **) indicate significant difference between calcium treatment and control at *P* < 0.05 and *P* < 0.01 level, respectively (t-Student test).

In Fig. 5, during a high incidence period of fruit creasing (from Oct. 15 to Nov. 15), the expression of *Cx*, *PE*, and *PG* were significantly higher in creased fruit pericarps than that in normal fruit pericarps, and this difference in *Cx* expression reached an extremely significant level (Fig. 5A). It has previously been reported that most of *Citrus* fruit creasing appear inside the canopy (Gambetta et al., 2000; Treeby et al., 2000). In this study, qRT-PCR was used to determine the expression levels of *PE*, *Cx*, and *PG* in the pericarps of exposed fruit peels and shaded fruit peels (Figs. 5B–5D). The expression levels of *PE* and *PG* were significantly lower in exposed fruit than that in shaded fruit on Oct. 15, and the expression of *Cx* in exposed fruit peels was significantly lower than in shaded fruit on Nov. 15. Moreover, the expression levels of *PE*, *Cx*, and *PG* were relatively stable in the exposed fruit peels, while larger fluctuations were observed in the shaded fruit from Oct. 15 to Dec. 1.

**Figure 6:**
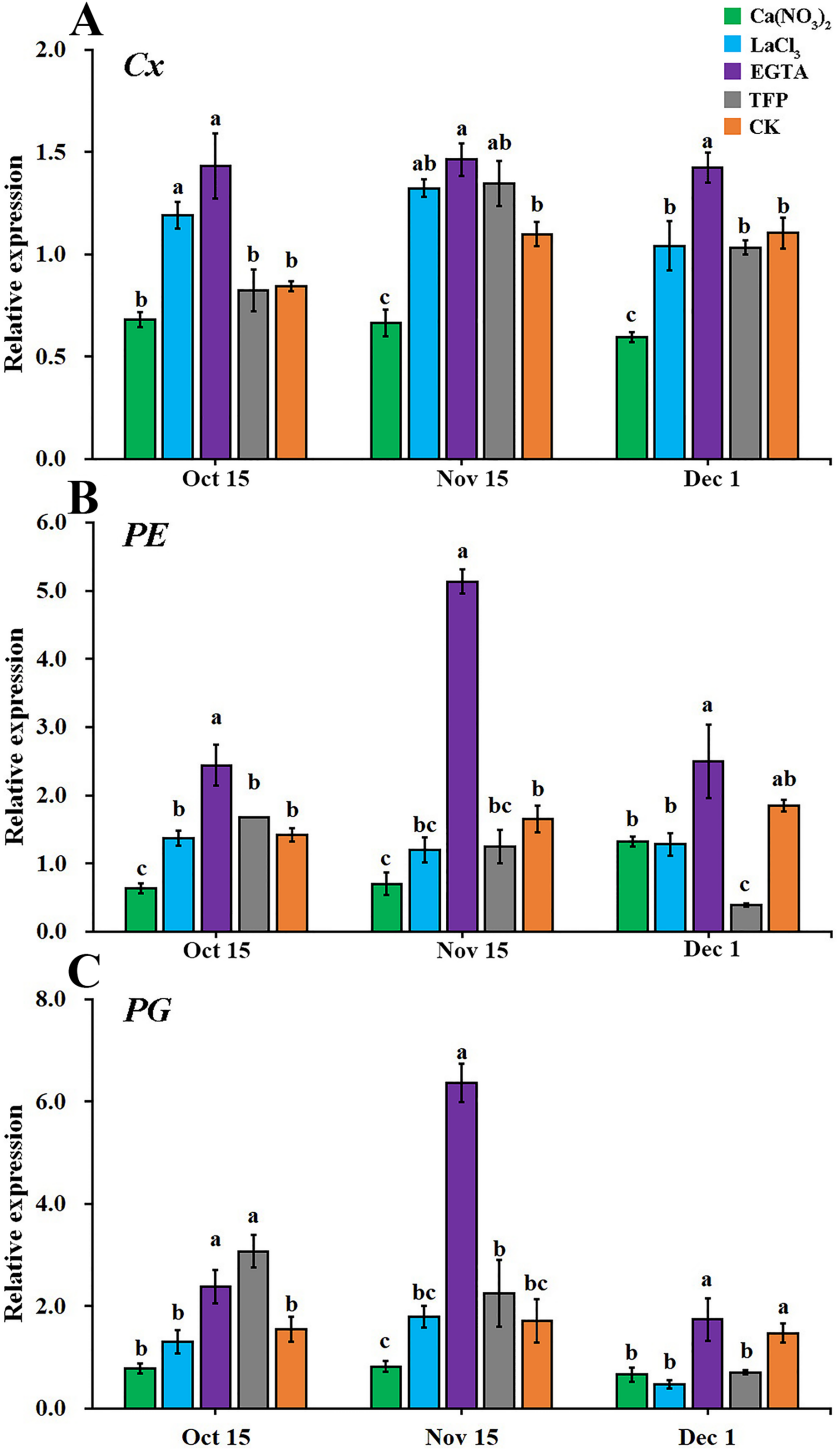
Effect of different treatments on the expression of metabolizing enzymes in ‘Hongjiang’ orange peel in 2014. (A) Effect of different treatments on the expression of *Cx* in ‘Hongjiang’ orange. (B) Effect of different treatments on the expression of *PE* in ‘Hongjiang’ orange. (C) Effect of different treatments on the expression of *PG* in ‘Hongjiang’ orange. The values are the means ± SEs (*n* = 3); Different lowercase letters indicate a significant difference between the treatments at *p* < 0.05 (Duncan’s multiple range test).

As shown in Fig. 6, the effects of the different treatments on the relative expression levels of the cell wall metabolic enzyme genes (*Cx*, *PE*, and *PG*) were similar. From the early stage of the occurrence of fruit creasing to the late stage, calcium nitrate treatment reduced the expression levels of *Cx*, *PE*, and *PG* the most in ‘Hongjiang’ orange peel. EGTA treatments increased the expression levels of *Cx*, *PE*, and *PG* in the pericarp. The expression of *Cx* was significantly lower in pericarps treated with calcium nitrate than that in the control on Nov. 15 and Dec. 1, while the expression of *Cx* in EGTA-treated peel was significantly higher than that of the control from Oct. 15 to Dec. 1. The expression of *Cx* in pericarps treated with LaCl_3_ was significantly higher than that of the control on Oct. 15 (Fig. 6A). *PE* expression was significantly lower in ‘Hongjiang’ orange peel treated with calcium nitrate than that in the control on Oct. 15 and Nov. 15, while its expression under the EGTA treatment was significantly higher compared with the control on Oct. 15 and Nov. 15. *PE* expression in peel treated with LaCl_3_ and TFP did not differ significantly from that of the control (Fig. 6B). The expression of *PG* was significantly lower in pericarps treated with calcium nitrate than in the control on Dec. 1, while its expression was significantly higher under the EGTA treatment and the TFP treatment than under the control on Nov. 15 and Oct. 15. The difference between the other treatments was not significant (Fig. 6C).

## Discussion

### Calcium nitrate application can reduce the incidence of creasing in ‘Hongjiang’ orange

Calcium is an essential element for plant growth and development, as well as a crucial component of cell walls (Hepler, 2005). Foliar Ca application led to decreases the fruit cracking in cherry (Winkler & Knoche, 2019), pomegranate (Davarpanah et al., 2018), watermelon (Lopez-Zaplana et al., 2020), grape (Yu et al., 2020) and *Citrus* (Chen et al., 2014; Wen & Shi, 2012). Thus, spraying calcium fertilizer is a common and effective agricultural measure to reduce fruit cracking. The goal of our study was to evaluate how calcium treatment affects *Citrus* fruit creasing or cracking and to elucidate the underlying physiological and molecular mechanism. This study suggested that the occurrence of creasing in ‘Hongjiang’ oranges began in middle and late September, and the peak period was from Oct. 15 to Nov. 15, which may be due to a decrease in calcium content in the later stages of rapid fruit growth (Winkler & Knoche, 2019). Thus, foliar application of calcium nitrate during fruit rapid enlargement significantly reduced the incidence of fruit creasing. The LaCl_3_ treatment (LaCl_3_, as a plasma membrane Ca^2+^ channel blocker, can compete with calcium ions for binding sites) and EGTA treatment produced the higher fruit creasing rate, which were significantly higher than that of the control. Previous study showed that EGTA and LaCl_3_ treatments can reduce the absorption of calcium by cells, resulting in Ca^2+^ deficiency and destroying the stability of cell walls (Liu, Yao & Han, 2014; Winkler & Knoche, 2019). Similar results had been obtained in two field experiments, and it is widely known that foliar sprays of calcium solutions could prevent fruit cracking (Davarpanah et al., 2018; Wen & Shi, 2012; Chen et al., 2014; Kafle et al., 2016; Bakeer, 2016; Michailidis et al., 2017; Yu et al., 2020; Michailidis et al., 2020, 2022). Calcium absorption increased by calcium treatment (Yu et al., 2020). The absorption efficiency of calcium is about 30% in apples (Kalcsits et al., 2017) and 77% in *Citrus* (Xiao et al., 1996) after calcium treatment. Thus, applying calcium directly to fruit is an effective method to supplement calcium nutrition. Taken together, Applying calcium fertilizer in the rapid expansion stage of ‘Hongjiang’ orange fruit can effectively reduce the occurrence of fruit creasing.

### Calcium may reduce fruit creasing by changing the mechanical properties of ‘Hongjiang’ orange peel

The pericarp plays an important role in fruit cracking resistance, transportability, storability and shelf-life quality (Wang et al., 2021). The thickness and hardness of fruit peel are important indicators to measure fruit strength, and they are directly related with citrus fruit peel creasing and cracking (Li & Chen, 2017). The fruit creasing rate on the shaded side of a tree or inside the tree canopy is higher than that on the sunny side or the outside (Treeby et al., 2000). Our research found that the thickness of exposed ‘Hongjiang’ oranges was higher than that of shaded oranges. The thicker the peel, the smaller the possibility of breakage, because epidermal cells are elongated and deformed to obtain greater elasticity of the peel and stronger resistance to cracking (Correia et al., 2018). Although the flesh and the peel develop inconsistently and the sudden expansion of the flesh is very rapid, the peel quickly adapts to this change to reduce the fruit cracking (Khalil & Aly, 2013; Li & Chen, 2017). Therefore, the difference in peel thickness is one of the main causes of the difference in the fruit creasing rates between exposed fruit and shaded fruit. Fruit ripening is a complex process involving many physiological and biochemical changes. As the *Citrus* fruit matures, it gradually softens. The present study found that ‘Hongjiang’ oranges gradually softened at the late stages of development, but there was no significant difference in the hardness of the fruit inside and outside the tree canopy. In the process of softening the *Citrus* fruit peel, uneven softening speed could result in poor appearance and even lead to the occurrence of fruit creasing (Li et al., 2009). Taken together, the mechanical properties of the pericarp are closely associated with fruit creasing or cracking in citrus fruit.

Mineral elements have been associated with improvement of the cracking resistance and firmness of fruits, the most important being calcium (Ca), boron (B), magnesium (Mg), potassium (K), and zinc (Zn). These are mainly associated with improvement of the cell membrane, cell walls or cuticles (Bakshi, 2018; Chater & Garner, 2018; Hardiyanto & Friyanti, 2019). we found that the Ca content in creased fruit peel was lower than that in the normal fruit peel. Similaritly, the Ca content in shaded fruit peel was lower than that in the exposed fruit peel. Thus, the occurrence of fruit creasing is closely related to the Ca content in the peel of ‘Hongjiang’ orange. Ca is used to improve fruit mechanical properties and to decrease the incidence of rots in kiwifruit and grape (Antunes et al., 2004; Ciccarese et al., 2013). And it can crosslink the cell wall constituents thereby altering the mechanical characteristics of the fruit skin and flesh (Brüggenwirth & Knoche, 2017). Also, calcium spray helped to increase the Ca content in the peel of ‘Hongjiang’ orange during the occurrence of fruit creasing. And correspondingly, calcium spray helped to increase the thickness of the ‘Hongjiang’ orange peel and increase the hardness in a certain degree. However, treatment with calcium inhibitors (LaCl_3_, TFP, and EGTA) showed the opposite effect.

Calcium is important for the structural integrity and stability of cell walls (Ranty et al., 2016) and is the richest mineral element in cell wall (Aghdam et al., 2012). Li et al. (2009) also showed that the hardness of fruit was closely related to the calcium content in the fruit. Foliar spraying calcium increases apple fruit firmness and prevents cell wall disassembly (Ortiz, Graell & Lara, 2011). Calcium treatment significantly delayed the increase in water-soluble pectins and the decrease in CDTA-soluble pectins and sodium carbonate-soluble pectins contents, which might contribute to stronger skin break force and higher resistance to fruit cracking (Yu et al., 2020). Calcium spraying could increases the calcium content of the peel (Davarpanah et al., 2018). It can crosslink the carboxyl groups of the homogalacturonan domain to form an ‘‘egg-box’’ structure which can strengthen cell wall (Yang et al., 2016; Shi et al., 2017). In conclusion, foliar spraying calcium can increase the Ca content in the peel resulting in changing the mechanical properties of ‘Hongjiang’ orange peel to reduce the fruit creasing and cracking rate.

### Calcium may reduce fruit cracking by inhibiting cell wall disassembly

Cell wall composition, modification, and disassembly can influence the fruit peel mechanical properties and may be an critical factor determining fruit susceptibility to cracking (Yang et al., 2016; Brüggenwirth & Knoche, 2017; Jiang et al., 2019). The cell wall component contain a network of interactions between polysaccharides, proteins and polyphenol compounds, which provide mechanical support and hardness for plant cells (Anderson & Kieber, 2020; Du, Anderson & Xiao, 2022). Fruit softening and cracking are closely associated with metabolism and biochemical modification of cell wall components, which are the result of the action of many enzymes, such as polygalacturonase (PG), pectinesterase (PE), cellulase (Cx) and expansin (EXP) (Brummell & Harpster, 2001; Miedes & Lorences, 2009). Among them, PE and PG can degrade the pectins. EXP, PG, β-Gal, and XET are closely related to fruit cracking in tomato, apple, litchi, and sweet cherry (Lu et al., 2006; Wang et al., 2006; Kasai et al., 2008; Balbontín et al., 2014). The activities of PG, Cx and β-Gal are significantly higher in a cracking-susceptible genotype in tomato fruit (Yang et al., 2016). Calcium nitrate application during rapid fruit expansion significantly reduced Cx activity in the pericarp during the occurrence period of fruit creasing, while the inhibitory effect on PG and PE enzyme activity was not significant. Both EGTA and LaCl_3_ application increased the activity of PG, Cx, and PE in the pericarp to some extent, which indirectly indicates that calcium has an inhibitory effect on cell wall degrading enzymes, because of EGTA extracting Ca from the cell wall (Winkler & Knoche, 2019). The activities of PG or Cx in the pericarps treated with TFP were significantly higher than that of the control on Sep. 15, and PE activity was significantly higher than that of the control on Dec. 1, indicating that CaM may be involved in the regulation of peel cell wall enzyme activity in ‘Hongjiang’ oranges. Although calcium nitrate application didn’t exhibit a better inhibition of cell wall hydrolase (Cx, PG and PE) activity, combined with the calcium inhibitor effect assay, adequate calcium content can reduced enzyme activity to some extent during the occurrence period of fruit creasing. The occurrence of fruit creasing is a fairly complex biological process with many genes involved. Li et al. (2014) found that there are 24 genes involved in cell wall modification, including 5 *LcPG* and 3 *LcPE* genes, which are expressed differently in normal lychee fruit and cracked lychee fruit. Schuch et al. (1991) reported that reducing the expression levels of *PE* and *PG* by antisense transgenic technology could reduce tomato fruit cracking. Our results suggested that the expression levels of *PG, Cx*, and *PE* were significantly higher in the peel of creased fruit than in the peel of normal fruit during the high-incidence period of ‘Hongjiang’ orange fruit creasing. The expression levels of *PG, Cx*, and *PE* were higher in the pericarps of shaded fruit than in the pericarps of exposed fruit. Moreover, the expression levels of *PG, Cx*, and *PE* were relatively stable in the pericarps of exposed fruit peels. We can conclude that the occurrence of creasing or cracking of ‘Hongjiang’ oranges maybe related to the high expression levels of the *PG, Cx* and *PE* genes in ‘Hongjiang’ orange peel. Dong et al. (2009) reported that foliar application of calcium significantly increased the calcium content in the flesh (80.05%) and peel (11.01%), and significantly reduced the expression of *PG, PE*, and *β-Gal* in the flesh, which was consistent with the enzyme alteration. And the hydrolysis of pectin was also inhibited. Yang et al. (2016) found that simultaneous suppression of SlPG and SlEXP1 expression in ripening fruits reduces cell wall disassembly and thereby reduces the fruit cracking rate by approximately 12%. Calcium treatment can inhibit the PME expression and PG to reduce the fruit cracking (Yu et al., 2020). Our study suggested that application of 0.5% Ca(NO_3_)_2_ significantly reduced the expression of *Cx*, *PE*, and *PG* in ‘Hongjiang’ orange peel. The application of EGTA significantly increased the expression of *Cx, PE*, and *PG* in ‘Hongjiang’ orange peel, while the effect of LaCl_3_ was not significant, which may be due to the mechanism of action of calcium on both. This indirectly proves that adequate Ca content in ‘Hongjiang’ orange peel has an inhibitory effect on the expression of *Cx* and *PG* in ‘Hongjiang’ orange peel, which reduces the fruit creasing or cracking rate.

## Conclusions

Foliar spraying of calciumnitrate at the fruit rapid enlargement stage can increase the Ca content in the peel of ‘Hongjiang’ orange, although its effect on changing the mechanical properties of pericarp and inhibiting the activity of hydrolase in the peel was not very significant in this study. It can significantly suppress the expression of cell wall degrading enzymes genes (*PG*, *PE* and *Cx*) in ‘Hongjiang’ orange peel during the high occurrence period of fruit creasing, thereby reducing the occurrence of fruit creasing and cracking. Thus, foliar spraying of calcium is an effective agricultural measure to reduce the fruit creasing or even cracking of ‘Hongjiang’ orange.

## Supplemental Information

10.7717/peerj.14574/supp-1 Supplemental Information 1 Raw data.

## Additional Information and Declarations

### Data Availability

The following information was supplied regarding data availability: The raw data is available in the Supplemental File.
